# Autophagy/Mitophagy in Airway Diseases: Impact of Oxidative Stress on Epithelial Cells

**DOI:** 10.3390/biom13081217

**Published:** 2023-08-04

**Authors:** Giusy Daniela Albano, Angela Marina Montalbano, Rosalia Gagliardo, Mirella Profita

**Affiliations:** Institute of Translational Pharmacology (IFT), National Research Council of Italy (CNR), Section of Palermo, Via Ugo La Malfa 153, 90146 Palermo, Italy; angelamarina.montalbano@cnr.it (A.M.M.); rosaliapaola.gagliardo@cnr.it (R.G.); mirella.profita@cnr.it (M.P.)

**Keywords:** oxidative stress, autophagy, mitophagy, lung disease, COPD, asthma

## Abstract

Autophagy is the key process by which the cell degrades parts of itself within the lysosomes. It maintains cell survival and homeostasis by removing molecules (particularly proteins), subcellular organelles, damaged cytoplasmic macromolecules, and by recycling the degradation products. The selective removal or degradation of mitochondria is a particular type of autophagy called mitophagy. Various forms of cellular stress (oxidative stress (OS), hypoxia, pathogen infections) affect autophagy by inducing free radicals and reactive oxygen species (ROS) formation to promote the antioxidant response. Dysfunctional mechanisms of autophagy have been found in different respiratory diseases such as chronic obstructive lung disease (COPD) and asthma, involving epithelial cells. Several existing clinically approved drugs may modulate autophagy to varying extents. However, these drugs are nonspecific and not currently utilized to manipulate autophagy in airway diseases. In this review, we provide an overview of different autophagic pathways with particular attention on the dysfunctional mechanisms of autophagy in the epithelial cells during asthma and COPD. Our aim is to further deepen and disclose the research in this direction to stimulate the develop of new and selective drugs to regulate autophagy for asthma and COPD treatment.

## 1. Introduction

Autophagy is implicated in different physiological and pathophysiological mechanisms in various tissues and organs and may be a target for new therapeutic approaches. It is involved in various diseases by its pleiotropic action (cancer, metabolic, neurodegenerative, cardiovascular, autoimmune, and pulmonary diseases) [1,2,3]. Autophagy represents an evolutionary mechanism involved in homeostasis that enables cell turnover by determining cell fate through a lysosome-dependent degradation pathway [1]. Autophagy mechanisms can be stimulated by multiple forms of cellular stress such as oxidative stress (OS), hypoxia, heat shock, hormonal imbalance, nutrient deprivation, chemical stress, and pathogen infections [4,5,6]. Autophagy is associated with reactive oxygen species (ROS)-mediated pathological responses in both cell signaling and cell damage. ROS produced by oxidative phosphorylation are highly reactive metabolites and can act as signaling molecules [7].

There are different autophagic pathways for the selective removal of mitochondria (mitophagy), proteasomes (proteaphagy), ribosomes (ribophagy), peroxisomes (pexophagy), endoplasmic reticulum (ER) (ER-phagy), lysosomes (lysophagy), lipid droplets (LDs) (lipophagy), and nuclei (nucleophagy) [8].

The main types of autophagic mechanisms are named macroautophagy, microautophagy, and chaperone-mediated autophagy. Although the autophagy pathways are morphologically distinct, all three culminate in the delivery of cargo to the lysosome for degradation and recycling of aged, damaged, and dysfunctional proteins and organelles. They are mechanistically different from one another in airway diseases [9].

Macroautophagy, referred to as “autophagy”, is responsible for organelle and microbial degradation. The macroautophagic process can be divided into four phases: (1) initiation (protein complexes are recruited to form autophagosome); (2) elongation (the assembly and stretching of the membranes takes place); (3) isolation (vesicular structures are generated to form autophagosomes); (4) termination (the intracytoplasmic content is closed in the double membrane vesicle to form the phagophore); (5) fusion (the autophagosomes fuse with the lysosome to transport the cytosolic material into the lysosomal lumen to perform the degradation by acid hydrolases, lipases, and cathepsins) [10,11,12].

Microautophagy refers to the degradation of cellular components largely without the formation of the autophagosome, but through lysosomal or endosomal membrane invagination, protrusion, or septation. Cytoplasmic elements are engulfed in autophagic tubes prior to fusion and degradation by lysosomal enzymes [13].

**Figure 1:**
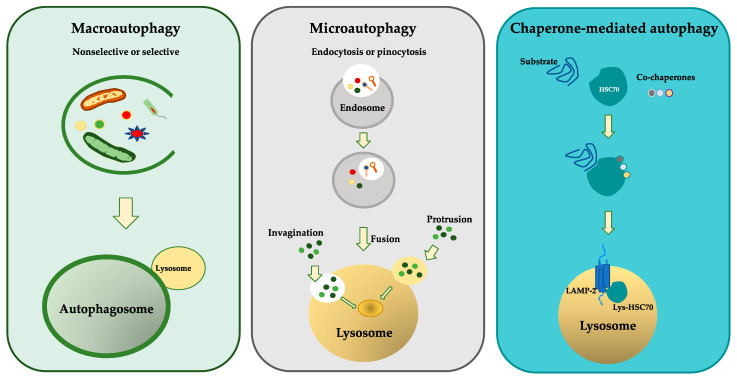
Overview of different autophagy pathways. The panels describe the three different types of autophagy including macroautophagy, microautophagy, and chaperone-mediated autophagy. Macroautophagy, through the formation of a double membrane vesicle surrounding the cytoplasmic cargo, forms an autophagosome which fuses with a lysosome, causing the degradation of its contents. Microautophagy induces invagination or fusion with a lysosome to degrade the cellular components. It engulfs cytoplasmic elements into autophagic tubes before fusion and degradation by lysosomal enzymes. Chaperone-mediated autophagy transports single unfolded proteins directly across the lysosomal membrane.

Chaperone-mediated autophagy (CMA) is a selective form of autophagy. It transports single unfolded proteins directly across the lysosomal membrane. The soluble proteins are selectively sequestered through a protein target complex called the HSC70 chaperone complex. Chaperones interact with other proteins to stabilize or help them to reach their native form and they are activated without being present in the final structure of lysosomes [14]. The HSC70 complex functions as a protein-folding catalyst and binds lysosome-associated membrane protein-2A (LAMP-2A) on the lysosomal membrane [14]. Thus, the target protein is translocated to lysosomes to be degraded [9] (Figure 1).

Autophagy pathways generate an accumulation of damaged proteins and organelles within the cytoplasm, giving rise to mitochondrial dysfunction, genomic instability, and ROS generation [7].

Under stress conditions, autophagy operates in different ways. Sometimes it efficiently removes ER, peroxisomes, and damaged mitochondria; in other cases, the cells can self-digest by supplying nutrients for protein synthesis and thus activating cell survival mechanisms [15]. Autophagy is regulated by a complex network of signaling cascades that can be separated into two categories of proteins. The first category is involved in the suppression of autophagy and includes proteins such as PI3 kinase proteins, Akt1, and anti-apoptotic family member BCL-2 [16]. The second category includes tumor suppressor proteins that activate autophagy (Beclin-1, Atg4c, BH-3, and PTEN) [16].

As previously mentioned, autophagy plays pleiotropic roles in different cell biological processes, such as metabolic regulation, cellular and tissue remodeling, response to pathogen invasion, antigen presentation, and in many other processes in various human diseases [16,17,18]. In particular, impaired mechanisms of autophagy are observed in the epithelial cells and the alveolar macrophages in several lung diseases including chronic obstructive pulmonary disease (COPD), asthma, idiopathic pulmonary fibrosis (IPF), interstitial lung disease (ILD), infectious lung disease, acute lung injury (ALI), and lung cancer [1,19].

## 2. Oxidative Stress and Dysfunctional Mechanisms of Autophagy/Mitophagy

Various noxae activate ROS production in the lung via different reactions. The exogenous noxae activate oxidases in neutrophils, macrophages, epithelial cells, and endothelial cells of the lung [20,21]. Although the subtle relationships are not yet clear, it is known that the interaction of ROS and the autophagic mechanisms are fundamental for the maintenance of cellular homeostasis. These relationships are dysregulated in the lung diseases [7]. Recent studies showed that autophagy is regulated by OS (especially by ROS) [7,22]. The definition of OS is “an imbalance between pro-oxidants and antioxidants with concomitant dysregulation of redox circuits and macromolecular damage” [23]. Increased levels of OS reduce the antioxidant defenses, affect the autophagy/mitophagy processes, and affect the regulatory mechanisms of cell survival in the lung epithelium [23]. They are involved in physiological and pathophysiological mechanisms that underlie many diseases, including pulmonary diseases [22,24].

OS leads to damage of proteins, lipids, and DNA and it induces the production of free radicals which are subsequently converted through enzymatic and non-enzymatic processes into ROS [25].

Among the ROS, there are oxygen-free radicals such as superoxide anions (O2^•−^) and hydroxyl radicals (•OH). These are very unstable molecules and can be oxidized by unpaired electrons. The O_2_^•−^ radical can react with NO to form a highly reactive peroxynitrite molecule (ONOO^−^) or be rapidly converted to hydrogen peroxidase (H_2_O_2_) by superoxide dismutase (SOD). H_2_O_2_ can be converted into the harmful ^•^OH in the presence of Fe^2+^ through the Fenton reaction. •OH can also generated from O_2_^•−^ via the Haber–Weiss reaction. In the presence of chloride (Cl^−^) and bromide (Br^−^) ions, H_2_O_2_ is catalyzed by heme peroxidase or myeloperoxidase to form hypochlorous acid (HOCl) and hypobromous acid (HOBr), known as very harmful oxidants [26].

The presence of low and controlled levels of ROS inside the cells allows the correct functioning of processes, such as protein phosphorylation, the activation of various transcription factors, apoptosis, immunity, and differentiation [27].

Cells deploy two antioxidant defense systems that are the first lines of defense against oxidants: the enzymatic antioxidant system including superoxide dismutase (SOD), catalase (CAT), glutathione peroxidase (GSH-Px), heme oxygenase-1, and small-molecular-weight redox proteins such as thioredoxins, peroxiredoxins, and glutaredoxins, and the non-enzymatic antioxidant system that includes vitamin C, vitamin E, and glutathione [23].

Enzymatic antioxidants are very efficient in promoting the reduction of H_2_O_2_ to H_2_O to limit its harmful effects [28]. Instead, non-enzymatic antioxidants work by interrupting free radical chain reactions [29]. To maintain redox homeostasis, oxidant and antioxidant systems antagonize each other to counteract the excess ROS, limiting the activation of autophagy mechanisms. Autophagy acts as a secondary defense and plays an important role against OS as the predominant synthesis of ROS is mediated by mitochondria [30]. Moreover, high levels of ROS activate mechanisms of mitochondrial dysfunction that are involved in the self-removal mechanisms by a process called mitophagy [23].

Autophagy is linked to the antioxidant response, and some transcribed proteins related to autophagy are regulated by redox status or OS. An example is the autophagy-encoded receptor p62, also called sequestosome 1 (SQSTM1) or autophagy-related gene 4b (ATG4b) (the main ATG4 protease for autophagy) [31]. p62 is involved in many signal transduction pathways, including the Keap1–Nrf2–ARE [Kelch-like ECH-associated protein 1–nuclear factor (erythroid 2-related factor 2)–antioxidant response element] pathway [32]. The Nrf2–Keap1–ARE pathway is a redox-sensitive signaling axis involved in the protection of cells from OS. p62 (an autophagy adaptor protein) enhances the dissociation of Keap-1 (the Nrf2 substrate adaptor for the Cul3 E3 ubiquitin ligase) from Nrf2 and promotes Keap1 degradation through p62-dependent autophagy, stimulating the antioxidant response [33,34,35,36]. Furthermore, p62 interacts directly with Nrf2, and the dysregulation of autophagy results in sustained p62-dependent activation of Nrf2 [33].

It has been demonstrated that the Keap1–p62 complex is recruited to autophagosomes by LC3 (light chain 3) and then degraded by autophagy [37]. Furthermore, p62 is involved in a positive feedback loop. In fact, under stress conditions, Nrf2 activation leads to its further increase by inducing p62 expression and thus maintaining its antioxidant effect [15,38,39].

## 3. Molecular Mechanisms of Autophagy

In mammals, autophagy and its mechanisms were discovered in the 1950s. However, only recently have 32 autophagy-related genes (Atgs) been identified. Sixteen Atg and two ubiquitin-like conjugation systems are involved in the formation of the autophagosome vesicle [15].

Autophagy induction is initiated by mammalian target of rapamycin (mTOR), a serine/threonine kinase that forms two different complex: Tor complex 1 and 2 (TORC1 and TORC2). TORC1 is mainly involved in regulating autophagy. It is activated upon nutrient deficiency or by 5′-AMP-activated protein kinase (AMPK) (a serine/threonine-protein kinase) upon high energy consumption. AMPK coordinates the induction of autophagy by inhibiting the mTORC1 target. Inhibition of mTORC causes the activation of the UNC51-like kinase (ULK) complex (composed of ULK1/2, Atg 13, FIP200, and Atg 101) that is the primary regulator of autophagy initiation. The activated ULK complex translocates to the ER forming a complex with various Atg proteins. Furthermore, activated ULK promotes the formation of the class III phosphatidylinositol-3-kinase complex (class III PI3K) composed of VPS34, VPS15, Beclin 1, Atg14L, and AMBRA1.

Beclin 1 is a protein involved in the regulation of autophagy. It is a key component of the PI3K complex that interacts with (1) several cofactors, such as Atg14, resistance to associated UV radiation (UVRAG), and Rubicon (regulator of the autophagosome size and number), or (2) Bcl-2 family proteins (autophagy inhibitors) [40,41]. Activation of the PI3K complex occurs by dissociation of Beclin1 from the anti-apoptotic proteins Bcl-2/xL. It promotes the conversion of PI to generate phosphatidylinositol-3-phosphate (PI3P) that is required for phagophore nucleation [9,42,43]. Furthermore, the PI3K complex contains vacuolar protein sorting 34 (VPS-34 kinase, encoded by PIK3C3) which phosphorylates and allows the nuclear translocation of transcription factor EB (TFEB), a master modulator of autophagy and lysosomal biogenesis, promoting its activation [44,45].

**Figure 2:**
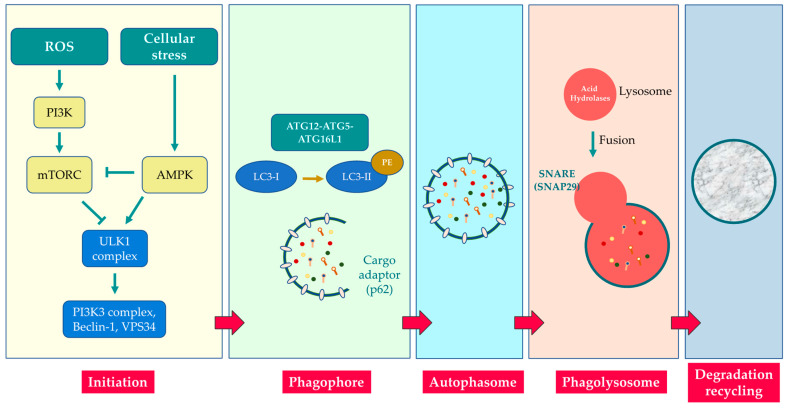
Autophagy pathway. ROS and cellular stress may inactivate mTORC1 through AMPK. Autophagy is negatively regulated by mTORC 1 inhibition that negatively regulates autophagy through direct phosphorylation of the ULK1 complex. The ULK-1 complex activates a PI3K class III complex including Beclin-1 phosphorylation and induction of VPS34 kinase activity, promoting the biogenesis of autophagosomes. The activation of the complex induces isolation membrane development, elongation, and recruitment of the Atg5–Atg12–Atg16–L1 complex that converts LC3-I to LC3-II through conjugation with phosphatidylethanolamine (PE). LC3-II binds p62 (an autophagy receptor) that links cargo proteins with the autophagosome membrane. In the final step, the autophagosome fuses with a lysosome through SNAP29 to form the autolysosome. Thus, lysosomal acid hydrolases degrade the autophagic cargo producing degradation products (such as amino acids) that are subsequently recycled back into the cytoplasm for reuse.

Subsequently, the phagophore elongates and closes to form the double-membrane autophagosome through the function of microtubule-associated light chain protein 3 (LC3-I). LC3-I is a ubiquitin-like protein and its conjugation with phosphatidylethanolamine (PE) converts LC3-I to LC3-II. Autophagosome maturation involves two distinct conjugations. The first involves the covalent bond between ATG 12 and ATG5. Subsequently, the Atg12–Atg5 complex forms a complex with Atg16 to act as an E3-like enzyme to form LC3-PE (LC3-II). LC3-PE (LC3-II) induces autophagosome closure by binding to the autophagosome membrane. This mechanism is mediated by Atg4-mediated proteolytic cleavage of LC3 [46,47]. SNAP29 is an autophagosome protein that interacts with certain molecules present on lysosomes to form the autolysosome. The content of the autolysosome (nutrients and amino acids) and LC3 are degraded by lysosomal acid hydrolases and released into the cytosol to be recycled [9] (Figure 2).

## 4. Molecular Mechanisms of Mitophagy

The autophagic mechanism that maintains mitochondrial homeostasis and removes aged and damaged mitochondria is called mitophagy [48]. Mitochondrial depolarization, hypoxia, and metabolic stress act as triggers for mitophagy, as well as the loss of the mitochondrial potential (Δψm). Mitochondrial depolarization induced by mitochondrial uncoupling agent carbonyl cyanide m-chlorophenyl hydrazone (CCCP) promotes the accumulation of PTEN-induced putative kinase 1 (PINK1, also known as PARK6) on the outer mitochondrial membrane, triggering mitochondrial degradation. There are different signaling mechanisms of mitophagy. The best characterized is PINK1–parkin RBR E3 ubiquitin protein ligase (Park-2)-dependent mitophagy [49].

**Figure 3:**
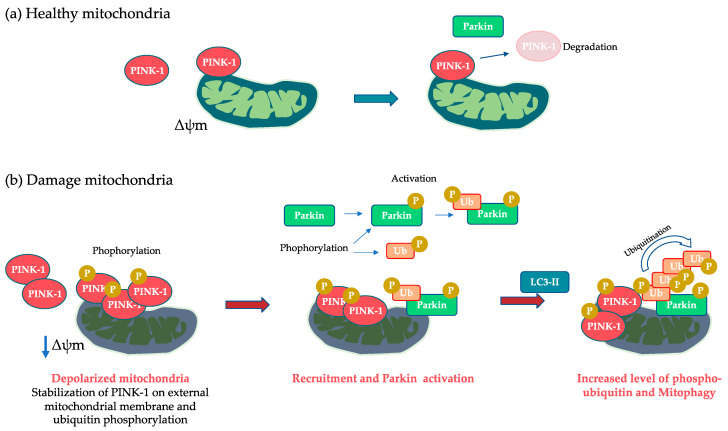
PINK1/Parkin-mediated mitophagy. (**a**) In healthy mitochondria, PINK1 does not accumulate on the external mitochondrial membrane; the protein is rapidly imported, processed, and degraded. (**b**) In damaged mitochondria following mitochondrial depolarization, PINK1 accumulates, leading to ubiquitin phosphorylation and consequent mitophagy and Parkin recruitment. Finally, activated Parkin promotes polyubiquitination which amplifies the signal for autophagy receptor recruitment and subsequent mitophagy.

PINK1 is a mitochondrial targeted kinase that interacts with different substrates to regulate mitochondrial functions, activate mitochondrial clearance, and participate in mitochondrial homeostasis [50,51]. Under normal conditions, PINK-1 translocates to the inner mitochondrial membrane where it is cleaved and subsequently degraded. However, if the mitochondria are damaged or polarized, it accumulates and autophosphorylates in the external membrane, inducing the (cytosolic) recruitment of Park-2 [9,52]. Park-2 is an E3-ubiquitin protein ligase that amplifies PINK-1-activated signaling that is involved in lysosomal degradation and the removal of mitochondria by autophagy [53,54]. Another signaling mechanism of mitophagy involves the BH3-only protein Bnip3 autophagy receptor through the interaction of its LC3-interacting region (LIR) with Atg8 proteins that act as mitophagy receptors [55] (Figure 3).

Furthermore, mitochondrial depolarization, hypoxia, and metabolic stress act as triggers for mitophagy, as well as loss of the mitochondrial potential (Δψm). Mitochondrial depolarization induced by mitochondrial uncoupling agent carbonyl cyanide m-chlorophenyl hydrazone (CCCP) promotes accumulation of PINK1 on the outer mitochondrial membrane and recruitment of Parkin to damaged mitochondria, thus triggering mitochondrial degradation [7,55,56].

Mitophagy can also be triggered by FUN14 Domain-Containing 1 (FUNDC1) protein located in the outer mitochondrial membrane. Phosphorylation of FUNDC1 occurs in response to different stress conditions, such as hypoxia or loss of the mitochondrial membrane potential, in key residues such as Ser13, Ser17, and Tyr18. This phosphorylation changes the binding affinity of FUNDC1 for LC3 and consequently affects mitophagy [57].

Another mechanism involves cardiolipin, a dimeric phospholipid of the inner mitochondrial membrane. Cardiolipin has a high content of unsaturated fatty acids, which gives it a high susceptibility to high levels of ROS and therefore acts as a trigger of mitophagy [58]. Cardiolipin can have different oxidation states that will induce cytoprotective mitophagy or stimulation of mitochondrial death pathways [59].

In the inner mitochondrial membrane, the protein Prohibitin 2 (PHB2) participates in the mechanisms of parkin-induced mitophagy, acting as a receptor that binds to LC3. PHB2 dysfunction interferes with the occurrence and development of various diseases including lung cancer [60].

Mitochondrial dysfunction and impaired mitophagy may contribute to the pathogenesis of many human diseases including lung disease. In particular, it affects the activity of airway epithelial cells in COPD and asthma [54,61].

## 5. Epithelial Cells in Airway Diseases

The respiratory epithelium is a complex system that is finely regulated and in direct contact with the external environment. It is stimulated by noxious stimuli such as microbes, allergens, cigarette smoke (CS), and air pollutants such as ozone or particulate matter (PM) [62]. The epithelium plays a key role in the transportation of gases to and from the alveoli, in the initiation and orchestration of pulmonary inflammation, immune responses, and tissue remodeling [63]. The epithelial cells are exposed to lesions and insults from both the external environment and endogenous signals (e.g., OS) that trigger DNA damage and ATP depletion [64].

The epithelial layer is formed by ciliated cells, mucus-producing goblet cells, club cells (Clara cells) as the dominant secretory cells, and basal cells as the key modulators of respiratory homeostasis. The basal cells are strongly attached to a basement membrane and to each other through tight junctions (TJs) and adherens junctions (AJs), which contribute to epithelial regeneration following injury. Under the basal membrane there is the lamina propria, covered by bands of airway smooth muscle cells, and formed by an extracellular matrix (ECM) mixture containing a variety of immune cells and fibroblasts [65,66].

The airway epithelium contributes to the host defenses through several mechanisms: (1) barrier function; (2) mucociliary clearance; (3) production of antimicrobial peptides (AMPs) and proteins; (4) production of ROS and nitrogen species (RNS); and (5) production of a multitude of cytokines, chemokines, and growth factors [67,68].

In the apicolateral portion of the epithelial cells, the barrier function is performed by the apical junctional complex formed by TJs, AJs, and desmosomes linked to the cytoskeleton, which maintain the structure of epithelial layer. Occludin, the claudin family, junctional adhesion molecule (JAM), and zonula occludens (ZO) are TJ proteins related to the actin cytoskeleton. They play a central role in paracellular permeability and epithelial polarity regulation [69,70,71]. E-cadherin is the main component of AJs. It is a transmembrane glycoprotein involved in the adhesion of epithelial cells to the cytoplasmic domain by the actin cytoskeleton [72]. Furthermore, the cytoplasmic domain binds β-catenin protein and avoids its translocation into nuclear regulatory pathways affecting proliferation, cell recognition, polarization, and cell migration [72,73]. Finally, desmosomes are intercellular junctions giving resistance to the epithelial barrier, and, in this way, control the entry of external noxae. They are involved in the regulation of epithelial permeability, gene expression, differentiation, apoptosis, cell proliferation, and immunological responses [74].

As previously mentioned, mucociliary clearance is among the innate defense mechanisms and mainly composed of mucus. Mucus is constituted by an extracellular gel composed of water, mucins, and numerous associated molecules. The key parameters for mucociliary clearance are mucus viscosity and ciliary function [68,75]. To evaluate the quality of the mucus, three fundamental parameters are taken into consideration: the mucin components, the hydro-ionic fluxes, and the rheological properties (physical properties of the mucus flow). The polymeric mucins mainly secreted in mammalian airways are MUC5AC and MUC5B, which differentially contribute to the pathogenesis of lung diseases [76,77].

Ciliary function can be assessed by correlating dynamic studies such as the cilia beating frequency or speed of propelling the mucus, and structural features such as cilia length and number of cilia per cell [78].

Thus, the respiratory epithelium is involved in maintaining pulmonary homeostasis. Dysfunctions in the bronchial epithelium, triggered by pathogenic noxae or environmental pollutants, induce increased epithelial permeability and consequent susceptibility to infections. This is the cause of the persistent inflammation that is characteristic of many respiratory diseases such as COPD, asthma, cystic fibrosis (CF), and lung cancer, among others [79,80,81,82].

Recently, several scientific groups found that autophagy mechanisms might play a pivotal role in the pathogenesis of chronic inflammatory lung diseases by promoting cytokine release, epithelial cell dysfunction, lung fibrosis, and airway remodeling [83,84,85].

## 6. COPD and Autophagy/Mitophagy

Chronic obstructive pulmonary disease (COPD) is caused by exposure to inhaled noxious particles, notably tobacco smoke and pollutants. It is one of the major causes of morbidity and mortality worldwide [86]. Chronic obstructive pulmonary disease (COPD), characterized by progressive and irreversible airflow limitation, is caused by airway obstruction and destruction of the lung parenchyma [87]. The continuous exposure of the airways to the external agents promotes inflammatory-oxidative stress that leads to the eventual irreversible damage of the lung parenchyma and alveolar walls [88]. This damage is involved in the progression to alveolar emphysema, the primary pathological clinical feature of COPD strongly related to the age of the patients [89].

The pathogenesis of COPD has been associated with an excessive increase in autophagy and mitophagy, which lead to the programmed cell death of epithelial cells and emphysema [90]. The activation of mitochondria-selective autophagy, namely the connection between mitophagy and other regulated forms of cell death, such as apoptosis and necroptosis, is a driving factor of the COPD phenotype and underscores its importance in normal lung homeostasis and pathogenesis [91]. Accordingly, it is possible to think that different or higher levels of autophagy might be associated with different phenotypes of COPD. Cigarette smoke (CS) is the major risk factor for airway inflammation in COPD subjects. Its negative effects in the lungs persist even after smoking cessation [92]. They are triggered inflammatory defense mechanisms for the successful maintenance of homeostasis within the respiratory system. In response to stimuli, the lung employs several defense mechanisms including those of the epithelial barrier, where airway epithelial cells lining the respiratory tract secrete numerous substances including mucins, lysozymes, defensins, siderophores, and nitric oxide; or mucociliary clearance is used as that primary innate defense mechanism, in which motile, ciliated epithelial cells eliminate particles and pathogens trapped in mucus from the airways. Macroautophagy/autophagy can be critical for inhibiting spontaneous inflammation and for the response of leukocytes to infection in the lung [92].

A lot of evidence shows that abnormalities in autophagy may contribute to the pathogenesis of chronic inflammatory diseases of the lung such as COPD and asthma, exerting a dual role. Stress, nutrient starvation, and inflammatory stimuli often trigger autophagy in the cells [93]. Persistent and unregulated autophagy especially affects the epithelial cells of the lung, promoting lung injury. Such cellular damage and stress, caused by dysregulated autophagy, drives lung inflammation and injury in COPD by affecting epithelial cell functions [92]. In fact, autophagy can play a protective as well as a pathogenic role in lung diseases [94,95,96,97]. The mechanisms of autophagy modulate various physiological processes in the cells, controlling key cellular events that remove damaged proteins, molecules, and subcellular organelles. In this manner, autophagy may play a fundamental role in cellular, tissue, and organismal homeostasis [98]. Therefore, disease states may be associated with autophagy dysregulation due to alterations in the multicellular biology of the organism [99]. The specialized functions of autophagy (selective autophagy, such as mitophagy) may directly contribute to the regulation of pathogenesis in pulmonary diseases. Under pathological conditions, dysregulation of redox homeostasis results in excessive generation of ROS, leading to OS and the associated oxidative damage to cellular components. However, this homeostatic regulation of cellular processes may escape to deal with excessive autophagic targets, leading to cell death. So, in most cases, induction of autophagy in response to stress acts as a cytoprotective mechanism in the lung [95,96,97]. Recent studies have shown that OS can accelerate aging by depleting stem cells, accumulating dysfunctional mitochondria, and decreasing autophagy, all of which generate additional OS [89]. Autophagy controls the accumulation of aggresome bodies that are primarily comprised of aggregated (misfolded or damaged) proteins which trigger chronic inflammatory/apoptotic responses leading to senescence and emphysema progression as showed in pre-clinical studies using both human lung cells and tissues [100,101,102].

These observations suggest that autophagy augmentation controls lung disease progression in COPD subjects with emphysema. Furthermore, it was found that changes in autophagy flux and aggresome pathology can serve as an early prognostic marker for predicting emphysema initiation or progression. Emerging data suggest that induced autophagy impairment via Regulator Transcription factor-EB (TFEB) regulates the action of cigarette smoke, playing a central role in the progression of COPD emphysema [103]. The precise and early detection of aggresome pathology can allow the timely assessment of disease severity in COPD–emphysema subjects for prognosis-based interventions. The use of drugs inducing autophagy might reduce alveolar damage and lung function decline, resulting in a decrease in the current mortality rates in COPD subjects with emphysema [104]. However, further studies might be necessary to clarify the potential pharmacological approach to control the mechanisms of autophagy in the treatment of COPD patients.

A number of studies exploring the roles of autophagy in pulmonary diseases have been reported [95,105,106]. These data suggest that autophagy and mitophagy can play both protective as well as detrimental roles in human pulmonary diseases, in a cell type-specific manner [107,108]. The autophagic mechanisms are important regulatory mechanism in the lungs and frequently affect the normal or pathological activities of epithelial cells [109]. Mitochondrial dysfunction has been extensively studied in the pathogenesis of chronic lung diseases, including COPD [110,111], but less explored in asthma.

Autophagy is an ATP-dependent process, and the final cell fate is, at least partly, dependent on the balance between the demand for substrate removal and cell affordability, which are limited by energy supply [112]. In fact, energy conditions play a significant role in facilitating different types of cell death [113]. If autophagy/mitophagy is over activated, substances and organelles responsible for energy supply are excessively degraded. Intracellular ATP levels are important in switching between programmed cell death (PCD) or necrosis mechanisms [114,115]. So, lung functions are highly dependent on energy supply and are sensitive to reductions in ATP levels (through the removal of mitochondria). Thus, autophagy/mitophagy might contribute to define a switch between PCD and necrosis, likely explaining the dual role of autophagy/mitophagy in COPD [93].

In contrast, activated autophagy can worsen inflammatory responses [116,117], induce mucus production, and disrupt mucociliary clearance (MCC), affecting the activity of epithelial cells [118,119]. Microtubule-associated protein 1 light chain (LC) 3 is an autophagosome molecule present in the gene expression profiles of COPD stage 2 versus stage 0 smokers, representing a potential molecular target in these inflammatory diseases [120]. In samples of lung tissues from COPD patients and in alveolar epithelial cells exposed to CSE, increased expression of LC3B-II was observed, leading to the Fas-mediated induction of apoptosis through the activation of autophagy [117]. Additionally, it was observed that cigarette smoke extract (CSE) markedly elevated the LC3-II/I ratio and upregulated inflammatory cytokines, including TNF-α, IL-6, and IL-8, in lung tissues of exposed mice [121]. In vitro, CSE increases autophagosome formation, as well as the LC3-II accumulation in epithelial cells. The silencing of LC3B inhibited autophagy and protected epithelial cells from CSE-induced apoptosis [122]. Peripheral lung tissues from patients with severe COPD showed an increase in p62, LC3, and aggresomes compared with age-matched non-smokers, suggesting an impairment of autophagy in COPD [121]. The increase in p62 is related to disease severity in peripheral lung tissues of COPD patients. It is strongly correlated with increased expression of LC3 and BICD1 [123]. Similarly, p62 is increased in alveolar macrophages from COPD patients and smokers. Furthermore, the numbers of autophagosomes and mitochondrial dysfunction are increased, with a flux of impaired autophagy in COPD [123]. In vitro, this is mimicked by exposure of alveolar macrophages to CSE, resulting in an accumulation of LC3, ubiquitinated proteins, and aggregates, and with reduced autophagic flux.

In vitro, human bronchial epithelial cells (HBEC) and A549 cells acutely exposed to cigarette smoke showed an accumulation of polyubiquitinated proteins, indicating impaired proteostasis associated with increased ROS generation and cellular necrosis [124]. Furthermore, it was observed that the activation of autophagy in response to smoke has harmful effects on epithelial cells [118,125,126,127]. Acute CS exposure only initiated moderate changes in autophagy, but with chronic exposures, autophagy flux was impaired [100,103]. CS-induced mitochondrial dysfunction and lack of mitophagy also combine to induce cellular senescence and the progression of COPD. CS increases autophagosome turnover (flux), inflammation, and mucus production [122] to promote epithelial cell death both in vitro and in vivo [128,129]. Mitophagy, as with autophagy, has a dual role in COPD [130,131]. Damaged mitochondria generate a series of cellular cascades, leading to lung damage in COPD by mitochondrial fragmentation, which induces mitophagy initiation. Parkin-dependent mitophagy serves as a protective strategy by removing fragmented mitochondria to prevent the spread of the damage in the mitochondrial network [132]. For example, CSE induced cytoplasmic p53 accumulation and Parkin interaction, thereby inhibiting Parkin translocation to damaged mitochondria. In addition, impaired Parkin translocation to damaged mitochondria was observed in the lungs of chronic smokers and patients with COPD [130]. Furthermore, inadequate mitophagy induced cellular senescence, which was attenuated by mitophagy restoration [130]. Likewise, CSE-induced mitophagy was inhibited by PINK1 and PARK2 knockdown, leading to cellular senescence in HBECs [133]. These data support that mitophagy confers lung protective effects in COPD or CS exposure. Moreover, CS also induces mitochondrial dysfunction in airway epithelial cells and stimulation of mitophagy, which result in cell death by necrosis (necroptosis) [127]. Most studies, however, indicate that autophagy mechanisms are impaired in COPD. Therefore, whether activated autophagy protects the lung or aggravates COPD progression needs further elucidation. These contradictory findings support the concept that that autophagy has a dual role in COPD pathogenesis that should be further elucidated.

## 7. Asthma and Autophagy/Mitophagy

Asthma is a chronic disease of the airway, often of an allergic nature, characterized by the complex interaction of airway obstruction, bronchial hyperresponsiveness (BHR), and airway inflammation, generating recurrent episodes of wheezing, coughing, chest tightness, and breathlessness. Epithelial injury, goblet cell hyperplasia, subepithelial layer thickening, airway smooth muscle hyperplasia, and angiogenesis promote structural changes or airway remodeling in the airways of asthma patients. T-helper 2 (Th2) and type 2 (T2) innate lymphoid cells (ILC2) orchestrate the inflammatory process in asthma through the secretion of Th2 cytokines (IL-4, IL-5, and IL-13), promoting eosinophilic inflammation in the airway mucosa [134]. Recently, it was demonstrated that inflammation and remodeling might be considered parallel aspects of the asthmatic process and not only a consequence of chronic airway inflammation. Furthermore, asthma can be distinguished into two main endotypes: T2-high, characterized by increased eosinophilic airway inflammation and T2-low that presents neutrophilic airway inflammation and exhibits increased resistance to steroids [135].

Autophagy plays an important role in the atopy and asthma pathogenesis. It is mainly involved in several key processes of asthma [136] with a detrimental or beneficial action, depending on the cell types involved [87]. It is mainly involved in several key processes of asthma pathogenesis [136] via the regulation of the innate and adaptive immune responses [137] and it may also promote interleukin (IL)-18 secretion from airway epithelial cells in response to outdoor allergens [138]. Autophagy plays different roles in asthma: it is increased in the eosinophilic inflammation and type 2 response, whereas it is decreased in the neutrophilic asthma phenotype [9]. Autophagy is also a regulator of TGF-β1-induced fibrogenesis [139] and its inhibition by ATG5 deletion or treatment with Baf-A1 or 3-MA decreases the fibrotic effect of TGF-β1 [139]. Furthermore, recent studies in a mouse model demonstrated that autophagy exacerbates the eosinophilic inflammation linked to a T2-high endotype [140].

In asthma and other inflammatory diseases characterized by high inter-individual variability, autophagy is evaluated on a case-by-case basis. Autophagy plays a prevalent role in a variety of airway remodeling processes [131]. In fact, there is a strong correlation between autophagy activation and airway remodeling in asthma, with a higher expression of Beclin 1 and Atg5 along with reduced p62 in asthmatics compared with non-asthmatics [141]. Similarly, Atg5 is correlated with reduced lung function and airway remodeling in patients with severe asthma [142].

Genetic mutations and single nucleotide polymorphism (SNPs) in autophagy genes are also associated with asthma. Human asthma-associated polymorphisms in Atg5 are correlated with reduced lung function [142] and with promotion of airway remodeling in patients with severe asthma, as well as in childhood asthma [142,143]. Still, despite some data indicate a link between atg5 polymorphisms and asthma severity, subsequent studies did not detect an association of Atg5 gene polymorphisms with asthma severity [144], suggesting the need to further clarify this pathological association. Human polymorphisms in Atg5 and Atg7 are not linked to susceptibility to or severity of asthma, but they are linked with neutrophilic inflammation in the sputum of asthmatic patients, suggesting a link to non-Th2 asthma [144]. Furthermore, genetic polymorphisms in Atg5 and Atg7 genes were linked to airway remodeling and impairment in respiratory system mechanics in individuals with pediatric and adult asthma [145,146,147].

Higher levels of LC3, ATG5, and autophagosome formation were observed in fibroblasts and epithelial cells from lung biopsies of asthmatic patients compared to healthy control subjects [146]. However, further studies reported higher levels of Atg5, Beclin-1, and p-62 in the epithelial cells from lung sections of the large airways of asthma patients than in large airway smooth muscles (ASMs) when compared with healthy controls without the activation of autophagy [138,148].

In vitro studies showed an increase in the activation of autophagy in epithelial cell cultures treated with allergens or other antigens, suggesting its potential role in a detrimental disease progression in asthma [134,144]. Increased levels of Atg5 protein expression were found in airway epithelial cells from patients with severe asthma and correlated well with subepithelial fibrosis and increased levels of collagen-1 expression [147]. Other in vitro studies showed that IL-13 stimulates goblet cell formation and mucin 5AC secretion by an increase in LC3-II/Atg5 autophagic flux in human airway epithelial cells [149].

Mitophagy is a normal physiological process during cell life and functions as surveillance system for the mitochondrial population, eliminating superfluous and/or impaired organelles [150]. Mitochondrial dysfunction induces the production of excessive ROS and secretion of various inflammatory cytokines and proteins, leading to the development of asthma [151]. Mitochondrial dysfunction also affects different cell populations (including alveolar epithelial cells (AECs), fibroblasts, and immune cells), promoting the fibrotic process [152] by stimulating AEC-derived cytokines, leading to activation of myofibroblasts [153].

**Figure 4:**
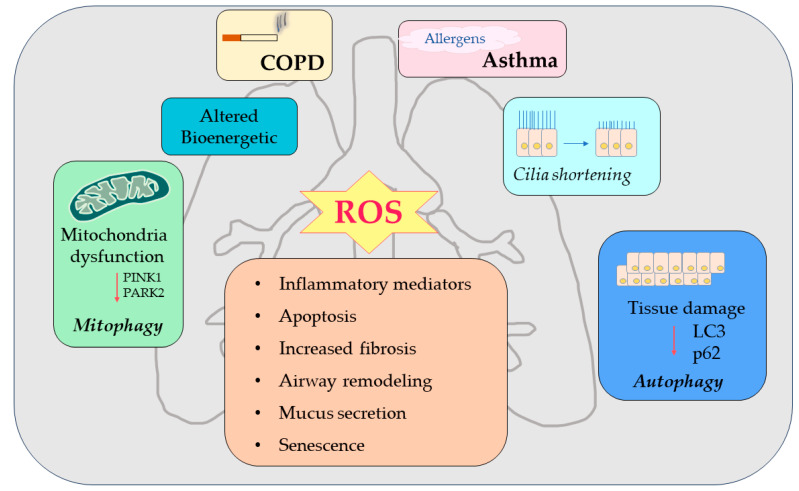
Dysregulated autophagy/mitophagy mechanisms in COPD and asthma. Exposure to the environment/cigarette smoke/allergen induces the generation of ROS in airway epithelial cells. These cells serve as “signaling molecules” that modulate the autophagic/mitophagic cycle process through the activation of signaling molecules and pathways. Dysregulation of autophagic and mitophagic mechanisms leads to progression of chronic inflammatory lung diseases such as COPD and asthma and to the onset of airway inflammation, airway remodeling, apoptosis, airway hyper-responsiveness, increased fibrosis, mucus secretion, and senescence.

The removal of damaged mitochondria is essential for the maintenance of cellular homeostasis; in fact, removal defects induce a greater activation of inflammatory pathways and the consequent establishment of chronic inflammation. Under physiological conditions, PINK-1 translocates to the IMM where it is cleaved and subsequently degraded [154]. Under challenged conditions, active PINK1 protein accumulates on the OMM through its interaction with the outer mitochondrial membrane complex (TOM complex), promoting Parkin recruitment through phosphorylation of both Parkin and ubiquitin [155]. In the nucleus, excessive production of ROS could activate HIF-1 FOXO3, and NRF2, promoting the transcription of BNIP33/NIX, LC3/BNIP3, and p62 to facilitate mitophagy [156]. Furthermore, the impairment of mitochondrial degradation by mitophagy can lead to the accumulation of fragmented mitochondria and activation of the mitochondrial apoptosis pathway [157]. It is well-documented that ROS are key mediators that contribute to oxidative damage and chronic airway inflammation in allergies and asthma [158,159,160,161]. All these studies suggest that the induction of ROS production in the lung might promote asthma pathogenesis through these mechanisms and through mitochondrial damage. However, there are limited studies that involved the description of the mechanism of mitophagy in asthma. Further extensive studies are needed to explore the underlying mechanism in terms of the mitochondrial dynamics and mitophagy regulation involved in asthma (Figure 4).

## 8. Pharmacological Approach in Airway Diseases

The control of autophagy and mitophagy might be considered as potential therapeutic targets to inhibit the pathogenetic mechanisms of diseases [151]. There is growing evidence that abnormal autophagy contributes to the pathophysiology of asthma and COPD and that pharmacological treatments might contribute to the restoration of autophagy by maintaining normal cell homeostasis. These benefits might help to control the disease. However, to date, no drugs specifically targeting the autophagy control have been developed and used. Furthermore, the role of autophagy in the pathogenesis of asthma and COPD is still uncertain, and it may reflect different responses in different cell types in response to different stimuli in in vitro studies, as well as in in vivo experimental models [9]. However, here, we report the most relevant data on the effect of some drugs in the control of autophagy in asthma and COPD.

Autophagy is reduced in several pathological conditions, and several drugs act as activators of the related mechanisms. They may be indicated in the treatment of patients with the phenotype of severe non-Th2 asthma or with stable COPD [162]. 3-methyladenine (3-MA) is an inhibitor of autophagy that acts by influencing the PI3K pathway. It suppresses the autophagosome formation in both asthma and COPD [136]. Rapamycin and metformin are autophagy activators; both attenuate AMP-activated protein kinase (AMPK), inhibit mTOR activation, and reduce the expression of proinflammatory mediators (IL-6 and CXCL8) in the endothelial cells of the lungs from COPD patients. Rapamycin and metformin regulate mitophagy and mitochondrial biogenesis to preserve energy metabolism in the cells [163,164]. One effect of rapamycin is to increase autophagy by reducing cell senescence, improving mitochondrial function [116].

The major risk factor for COPD is cigarette smoke. Some data obtained from in vitro and in vivo studies (COPD patients) showed that cigarette smoke affects the reduction in expression of the serine/threonine protein kinase mTORC1 in airway epithelial cells. Inhibition of mTORC1 not only activates autophagy but also inhibits the development of cellular senescence, improves mitochondrial function, and reduces protein synthesis and cell proliferation [165] and migration along with aberrant metabolic pathways [166]. However, there are no reports regarding a pharmacological approach using mTORC1 inhibitors in clinical studies of patients with lung diseases [167].

Carbamazepine is an anticonvulsant involved in the activation of the mechanisms of autophagy through the inhibition of PI3K signaling [168]. It inhibits the accumulation of aggresomes (insoluble clusters of ubiquitinated proteins that are formed as a cellular response mechanism to decreased proteasomal activity) in the lungs of mice exposed to cigarette smoke, preventing emphysema development [102]. However, there are no reports of the application of carbamazepine in the treatment of COPD in clinical studies.

Chloroquine and hydroxychloroquine inhibit autophagy by blocking lysosomal function. However, these drugs are nonspecific since they have numerous pharmacological activities. Therefore, their use as pharmacological approach in the lung is not appropriate [164]. A peptide derived from a region of beclin1 is named Tat-beclin1. It is a potent inducer of autophagy; it stimulates cell death and decreases protein aggregates in vitro. The potential therapeutic effect of the Tat-beclin1 peptide has been explored as a candidate therapeutic to induce autophagy in COPD patients [7]. The increased circulating Beclin1 levels accelerated aging markers in COPD [169]. These data suggest that Tat-beclin1 might be useful in controlling the complex mechanisms linking defective autophagy and cellular senescence in the progressive pathogenesis of COPD. Other potential regulators of the autophagy pathway could be 3, 4, 5-trihydroxyhexanostyrene (resveratrol) and oleuropein, two natural polyphenolic compounds found in grapes and olive oil, respectively. They are natural antioxidants that reduce CS-induced OS. In fact, they might have potent anti-inflammatory and antioxidant functions, and might inhibit autophagic dysfunction to improve the prognosis of COPD patients [19,170,171,172]. The treatment of epithelial cells with Quercetogetin has been proven to inhibit CSE-induced mitophagy and cell death by reducing the phospho-DRP-1 levels, thus suppressing apoptosis [173]. Furthermore, Puerarin promotes the inhibition of FUNDC1-mediated mitophagy through the activation of the PI3K/AKT/mTOR signaling pathway and reducing CSE-induced apoptosis in human epithelial cells [174]. Lastly, the phosphodiesterase 4 inhibitor Roflumilast has been found to protect epithelial cells from CS-induced mitophagy-dependent cell death [175]. Drugs currently used in the treatment of asthma such as dexamethasone, montelukast, and anti-IL-5 and anti-IgE antibodies are involved in the inhibition of autophagy [136]. However, since these drugs are not specific for autophagy treatment, they can play a dual role in the modulation of autophagy.

It was observed that statins can have a beneficial effect in patients with asthma, improving the symptoms and inflammation in asthma control [176]. However, the mechanisms of statin action are not clear. For examples, atorvastatin is a statin that showed the capacity to induce or inhibit excess autophagy [177]. It was observed that statins have a beneficial effect in non-Th2 smoking asthmatic patients [178], although it is not clear whether this benefit is mediated through increased autophagy. Furthermore, statins enhance the anti-inflammatory capacity of inhaled corticosteroids in asthmatic patients through effects on the mechanisms of autophagy [179]. Finally, statins can have beneficial effects by attenuating the neutrophilic inflammation in patients with COPD [180] (Table 1).

Activators (such as the mTOR inhibitor rapamycin) and inhibitors (chloroquine/hydroxychloroquine) of autophagy have been evaluated in the treatment of cancer [181,182]. However, few compounds have been considered to control the processes of autophagy in clinical applications. New therapeutic approach for lung disease treatment might include combinations of antioxidants or autophagy inhibitors/activators.

## 9. Conclusions and Perspectives

Globally, chronic inflammatory lung diseases are steadily increasing in adults and children and are among the leading causes of mortality. Autophagy represents an evolutionary mechanism involved in homeostasis, and mitochondria are the most intricate and dynamically responsive sensing systems of the cell. Specific signatures and mechanisms of autophagy/mitochondrial dysfunction are associated with lung disorder pathophysiology and clinical phenotypes that are becoming increasingly important.

Recent studies have demonstrated that restoring the normal physiological values related to autophagy can lead to a benefit in the progression of respiratory diseases such as COPD and asthma. However, this great potential does not yet exist as drugs that are specific for the modulation of autophagy despite regulating its mechanisms. Indeed, drugs such as rapamycin, dexamethasone, statins, and others are used to treat COPD and asthma, but they were not developed for the purpose of treating autophagic dysfunction.

This review aims to bring together the knowledge obtained to date to have a brief but effective glimpse of what we know and how we can use it to find new effective therapeutic targets.

## Figures and Tables

**Table 1:** Potential autophagy-related pharmacological treatments.

DRUG	Autophagy Target
3-methyladenine (3-MA)	PI3K inhibition
Rapamicyn	mTORC1 inhibition
Metformin	mTORC1 inhibition
Carbamazepine	PI3K inhibition
Chloroquine and hydroxychloroquine	inhibitor of toll-like receptors
Hydroxychloroquine	inhibitor of toll-like receptors
Tat-beclin1	PI3K CIII complex
Resveratrol	AMPK activation
Oleuropein	AMPK activation
Quercetogetin	phospho-DRP-1 inhibition
Puerarin	FUNDC1 inhibition
Statin	Not clear

## Data Availability

Not applicable.
